# From Seizure to Cerebral Actinomycosis: A Deceptive Case and an Unexpected Diagnosis Behind a Pseudotumoral Lesion

**DOI:** 10.7759/cureus.93468

**Published:** 2025-09-29

**Authors:** Salma Taouihar, Amine Bouabdallaoui, Ibrahim Kribou, Soukaina Wakrim, Hicham Nassik

**Affiliations:** 1 Department of Anesthesia and Critical Care, Souss Massa University Hospital, Faculty of Medicine and Pharmacy, Ibn Zohr University, Agadir, MAR; 2 Department of Radiology, Souss Massa University Hospital, Faculty of Medicine and Pharmacy, Ibn Zohr University, Agadir, MAR

**Keywords:** brain abscess, cerebral actinomycosis, craniotomy, pseudotumoral lesion, seizures

## Abstract

Cerebral actinomycosis is a rare bacterial infection of the central nervous system, often under-recognized due to its non-specific clinical and radiological presentation, which can mimic a tumor or other infectious process.

We report the case of a 53-year-old man with no significant medical history who was admitted for status epilepticus preceded by headaches. Brain imaging (CT and MRI) revealed multiple loculated frontal lesions with surrounding edema and mass effect, suggestive of a pseudotumoral or aggressive infectious process. Despite investigations and empirical antibiotic therapy, the lack of improvement led to a craniotomy with abscess drainage. Histopathological analysis confirmed the diagnosis of cerebral actinomycosis. The patient showed a favorable outcome under third-generation cephalosporin therapy.

This case highlights the importance of including cerebral actinomycosis in the differential diagnosis of brain abscesses and pseudotumoral lesions, even in patients without obvious risk factors. Diagnosis relies on histopathological examination, and management requires a combination of surgical intervention and prolonged antibiotic therapy. Clinical vigilance is crucial for early diagnosis and treatment, thereby improving prognosis.

## Introduction

Actinomycosis is a chronic, slowly progressive bacterial infection caused by filamentous, Gram-positive bacilli of the genus Actinomyces, which are commensal organisms of the oral, digestive, and genital mucosa. Although it most commonly affects the cervicofacial, thoracic, or abdominal regions, central nervous system (CNS) involvement is rare, accounting for less than 5% of cases [1,2]. Its clinical presentation is often misleading, mimicking tumoral, vascular, or other infectious processes, which makes high clinical suspicion essential for timely diagnosis [1,3].

Diagnosis primarily relies on histopathological analysis or culture, which can be challenging due to the bacterium’s slow growth and strict anaerobic requirements. Early recognition is crucial, as it allows for the initiation of combined surgical and antibiotic therapy, which has been shown to significantly improve patient outcomes [2,4,5]. Furthermore, long-term follow-up is recommended to monitor for recurrence, given the chronic and insidious nature of cerebral actinomycosis [3,5].

This case report emphasizes the need to consider cerebral actinomycosis in the differential diagnosis of brain abscesses and pseudotumoral lesions, particularly in patients with predisposing factors such as immunosuppression or poor oral hygiene, and underscores the impact of prompt diagnosis and multidisciplinary management on prognosis [2,3,5].

## Case presentation

A 53-year-old man, with no relevant medical, surgical, or infectious history (no head trauma, sinusitis, or recent dental extraction), and with an unremarkable immune status, including a negative HIV serology, was admitted to the emergency department for generalized tonic-clonic seizures occurring in the context of a six-day history of headaches. The seizure episode was refractory to initial antiepileptic treatment, prompting transfer to the intensive care unit for status epilepticus.

Upon admission, the patient was unconscious with a Glasgow Coma Scale score of 7 (eye opening: 3; verbal response: 1; motor response: 3), febrile at 38.5 °C, and presented with right-sided hemiparesis. Vital signs showed a blood pressure of 120/65 mmHg, a heart rate of 109 beats per minute, and an oxygen saturation of 98% on a 2 L/min nasal cannula oxygen. The clinical examination was unremarkable, apart from poor oral hygiene and multiple dental caries. Transcranial Doppler ultrasound showed a pulsatility index (PI) of 1.55 on the left (diastolic velocity: 24.7 cm/s) and 1.7 on the right (diastolic velocity: 29.3 cm/s).

**Figure 1:**
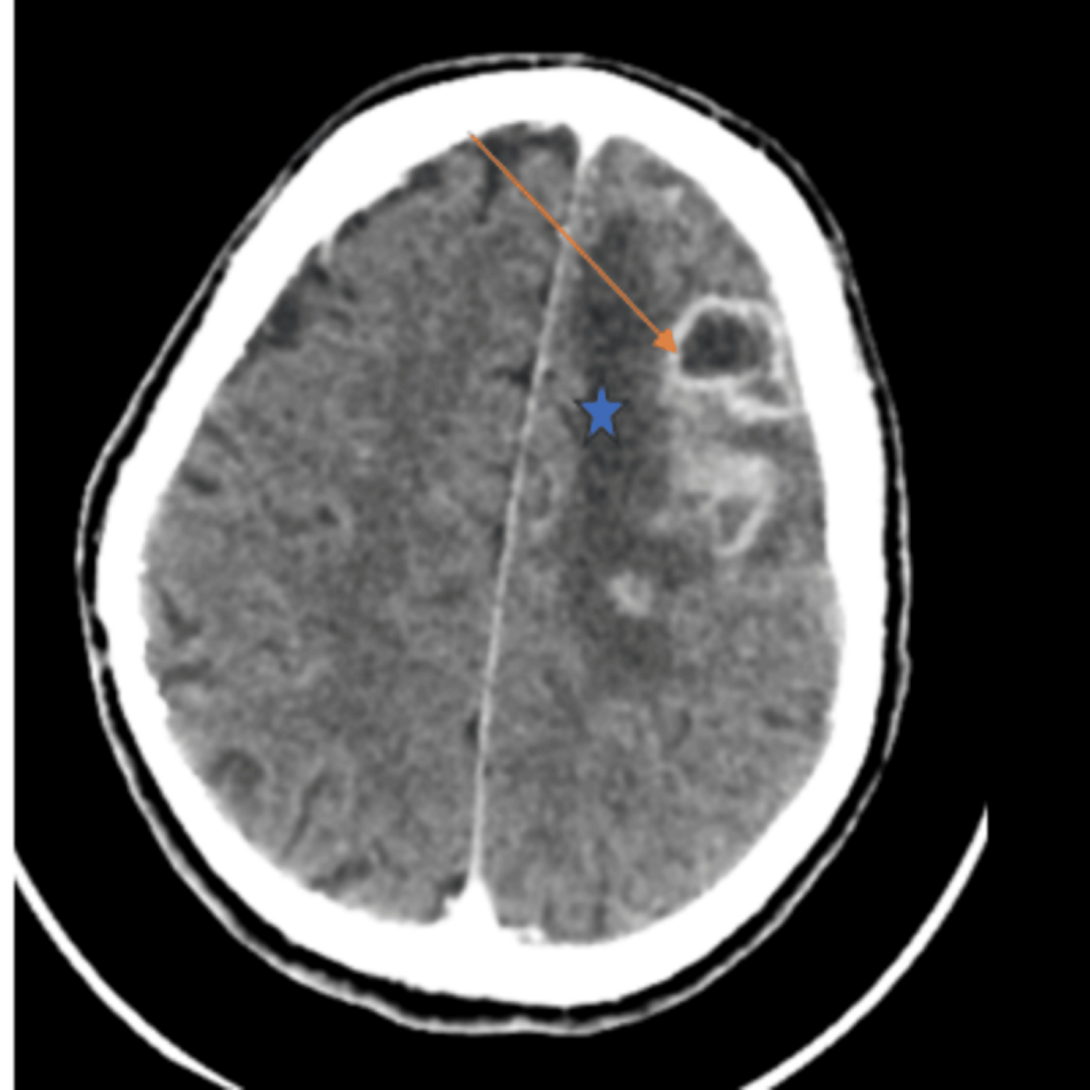
Cerebral CT images after injection of contrast showing left fronto-cortical and subcortical lesion (orange arrow) and peri-lesional edema (star)

Initial brain CT revealed a left fronto-cortical and subcortical lesion measuring 26 × 39 × 20 mm, with subfalcine herniation, initially suspected to be of tumoral origin (Figure 1).

**Figure 2:**
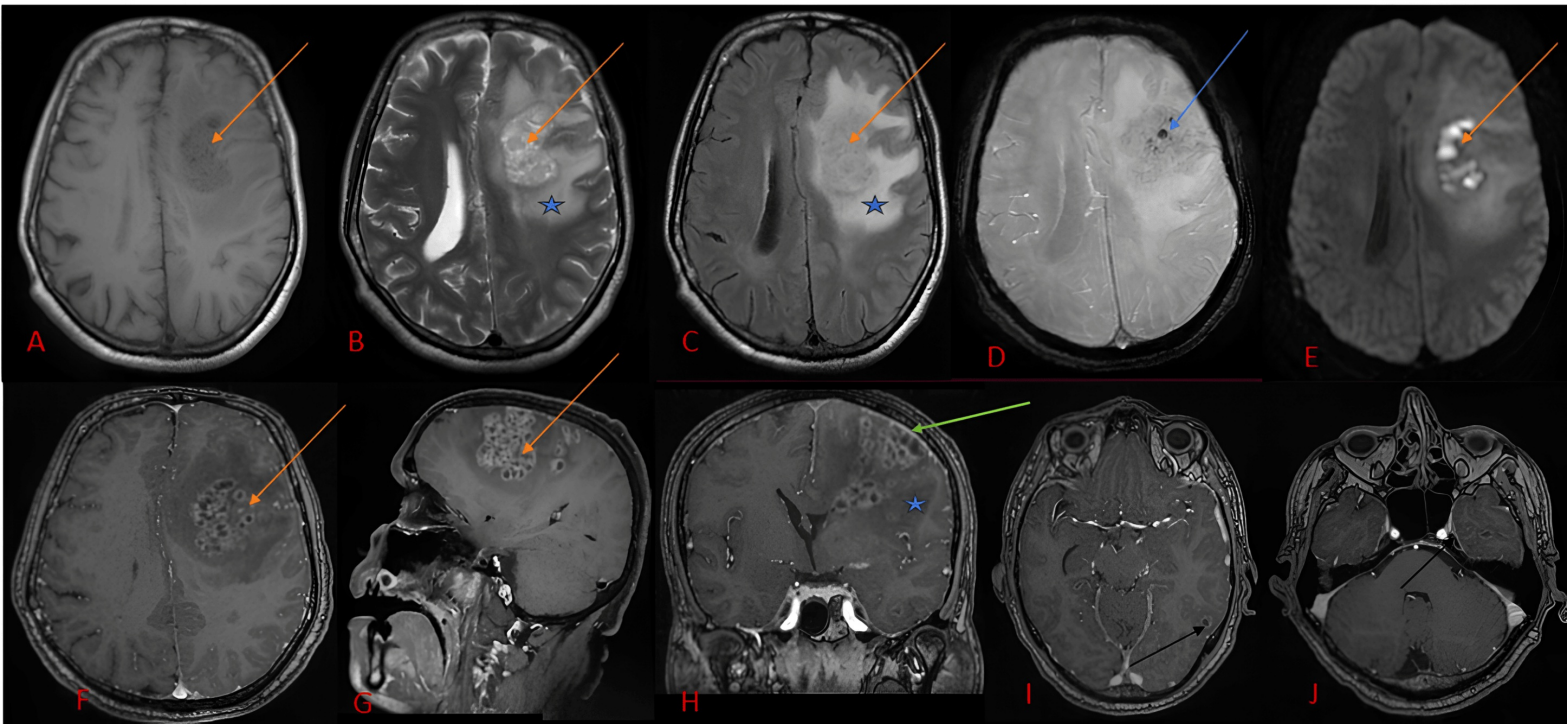
MRI images showing a multi-loculated left frontal lesion (orange arrow) with log contents in T1 hypo signal (A), intermediate signal in T2 and FLAIR (B, C), with diffusion restriction (D), and gadolinium enhancement of the log walls (F, G), with hemorrhagic remodeling in SWI (E) signal void (blue arrow), with individualization of other similar lesions at a distance (black arrow in I, J), peri-lesional edema (star B, C, and H) and a nearby meningeal reaction (green arrow in H) FLAIR: fluid-attenuated inversion recovery; SWI: susceptibility weighted imaging

Subsequent brain MRI showed multiple supratentorial, loculated, bilateral, and asymmetric lesions, predominantly in the left frontal lobe, associated with significant perilesional edema and mass effect, suggestive of an aggressive infectious process of bacterial or fungal origin (Figure 2).

Biological work-up revealed leukocytosis at 16,440/mm³ and an elevated C-reactive protein (CRP) level of 109 mg/L. The remainder of the standard laboratory tests were within normal limits. Lumbar puncture showed 29 white blood cells/mm³ with a lymphocytic predominance, decreased cerebrospinal fluid (CSF) glucose at 0.75 g/L (with concomitant blood glucose of 1.97 g/L), and elevated CSF protein at 1.85 g/L. CSF bacterial PCR, *Mycobacterium tuberculosis* testing, and serologies for HIV, syphilis, and toxoplasmosis were all negative.

The patient was intubated due to altered consciousness and intracranial hypertension, placed on controlled mechanical ventilation, deeply sedated, and started on norepinephrine to optimize cerebral perfusion. Empirical treatment was initiated, consisting of ceftriaxone, acyclovir, first-line antitubercular therapy, methylprednisolone, and antiepileptic therapy.

**Figure 3:**
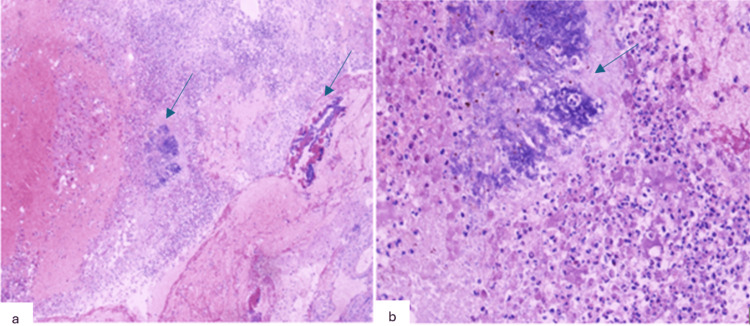
(a) Moderately to markedly intense polymorphous inflammatory infiltrate rich in neutrophilic polymorphonuclear cells, (b) focal infiltrates surrounding a few basophilic, fibrillary grains with an actinomycotic appearance

Given the persistence of fever and the patient’s clinical stabilization, a craniotomy with drainage of the cerebral abscess and biopsy of the left frontal lesion was indicated and optimally performed within 48 hours of admission, once the patient was hemodynamically stabilized. Intraoperative exploration revealed an encapsulated lesion containing purulent material, which was completely drained, and the capsule was evacuated. Histopathological analysis showed altered cerebral parenchyma with acute suppurative inflammatory infiltrates, containing focal clusters of basophilic organisms consistent with actinomycosis (Figure 3).

Following diagnostic confirmation, an extension work-up was performed, including thoracic computed tomography, abdominal ultrasound, and transthoracic echocardiography, which revealed no extracerebral infectious focus. No predisposing factors were identified, apart from poor oral hygiene.

Regarding clinical evolution, sedation was discontinued after the patient left the operating room. A tracheotomy was performed on day 6 of hospitalization, and on day 20, a silver tracheostomy tube was placed to facilitate the weaning phase before full decannulation. Neurological improvement was favorable, with progressive recovery of consciousness, although right-sided hemiparesis persisted as a sequela.

Once cerebral actinomycosis was confirmed, antiviral therapy (acyclovir) and antitubercular treatment (ERIPK4) were discontinued. Third-generation cephalosporin therapy was maintained with ceftriaxone at a dosage of 2g IV twice daily for six weeks, followed by an oral step-down with high-dose amoxicillin for an additional six months. The total duration of therapy will be adjusted based on clinical and radiological evolution under the supervision of infectious disease specialists.

The patient’s clinical course was favorable, allowing transfer to the infectious diseases department for continuation of targeted antibiotic therapy and long-term follow-up. At discharge, the Glasgow Coma Scale score had fully recovered to 15. Neurological examination demonstrated partial improvement of motor strength on the previously affected side, with only mild residual weakness. The patient was able to ambulate independently with minimal assistance, and a structured rehabilitation plan was arranged to support ongoing functional recovery.

## Discussion

Actinomycosis, a rare yet curable chronic bacterial infection, originates from Gram-positive bacilli of the *Actinomyces *genus, naturally colonizing the gastrointestinal and genitourinary tracts. Pathogenicity arises when a breach in the mucosal barrier allows these bacteria to spread across anatomical structures [1,2]. Although found worldwide, recent epidemiological studies suggest a slight increase in reported cases, likely attributable to enhanced diagnostic techniques and greater awareness; however, it remains an uncommon infection [2]. In the most recent systematic review (1988-2022, 118 cases), a mean patient age of 44 ± 20.6 years was reported, highlighting its rarity in very young children and individuals over 60, and a male predominance of approximately 57% [1,2] was noted. Geographically, most published cases originated from Asia (49.2%), followed by the Americas (28%) and Europe (20.3%). Despite diagnostic advancements, challenges in identification persist, as underscored by the 2023 systematic review [2].

The cervicofacial form is the most frequent (60% of cases), followed by the abdominal form (20%) and the pulmonary form (15-20%) [1,3]. Involvement of the central nervous system (CNS) is exceptional [5-7]. When it does occur, it most commonly presents as brain abscesses (67%), but may also manifest as meningitis (13%), actinomycetomas (7%), subdural empyemas (6%), or epidural abscesses (6%) [1,3]. CNS involvement is usually secondary to hematogenous dissemination or direct extension from ENT sources. Risk factors include cranial trauma, chronic ENT infections (such as sinusitis or mastoiditis), and surgical interventions [1,8]. Actinomycosis may affect various organs and mimic tumors or other chronic infections, particularly tuberculosis, especially in its cervicofacial forms [1,8]. Clinical presentation is variable, ranging from isolated headaches to focal neurological deficits, depending on the lesion’s location [2,3]. Diagnosis relies on identifying the organism in pus or tissue samples; however, isolation is often difficult (<50% of cases) due to prior antibiotic use or improper sampling [1].

Culture requires anaerobic conditions and may take between five and 20 days. In addition, recent literature highlights the growing use of metagenomic next-generation sequencing (NGS) to identify *Actinomyces* when cultures are negative or delayed; recent cases document diagnoses achieved by NGS subsequently confirmed by biopsy [1,3]. Imaging studies (CT or MRI), although non-specific, are valuable for assessing the extent of infection [1-4].

CNS actinomycosis typically presents with symptoms that resemble those of other pyogenic infections, with focal neurological deficits and signs of intracranial hypertension in the absence of meningitis [1-3]. The specific symptomatology is closely linked to the anatomical location of the lesions. Fever is inconsistent, occurring in fewer than 50% of cases, which complicates diagnosis and may lead to initial suspicion of a brain tumor [1-3]. Actinomycotic meningitis, less frequently observed, usually results from the rupture of a parameningeal focus into the subarachnoid space. It may present as acute bacterial meningitis or evolve into a chronic form, further complicating the diagnostic process [1,5].

The disease typically has an insidious course, with slower development of abscesses and empyemas compared to classical bacterial infections [2,3]. This chronic phase may be punctuated by acute neurological decompensation, especially in cases of intraventricular or subarachnoid rupture [2]. Temporary spontaneous remissions followed by neurological relapses are also frequently reported during the clinical course [5,9,10]. The temporal and frontal lobes are the brain regions most commonly affected [6].

Microscopically, cerebral actinomycotic abscesses appear as encapsulated purulent collections surrounded by a granulation zone rich in plasma cells, monocytes, and lymphocytes, often associated with edema and vascular congestion. Characteristic granules are frequently observed within these lesions [1,2,4,6]. Definitive diagnosis relies on the bacteriological isolation of *Actinomyces*, though this is often challenging. Histological examination may strongly support the diagnosis by revealing actinomycotic granules, which are clusters of filamentous bacteria visible under microscopy or even macroscopically. On hematoxylin and eosin staining, they appear as round or oval basophilic structures with radially arranged eosinophilic filaments [2,4,6]. However, histological diagnosis may be limited by the sparse number of granules in the tissue [3].

Brain imaging is essential to evaluate the extent of lesions, but it cannot differentiate actinomycosis from other infectious or non-infectious conditions. CT imaging typically shows thick-walled, ring-enhancing, irregular or nodular lesions, corresponding to cerebral or cerebellar abscesses, most often solitary but sometimes multiple, often associated with granulomatous inflammation. These lesions exhibit homogeneous enhancement, thickened walls, and surrounding edema [2,3]. MRI is more sensitive for detecting involvement of the subdural space, cavernous sinus, or internal auditory canal, especially in cases of acute purulent meningitis. Magnetic resonance spectroscopy may show elevated levels of amino acids, acetate, and succinate, although these findings are non-specific [2,4,6].

Therapeutic management relies on the same principles as for other intracranial pyogenic infections, namely effective surgical drainage combined with prolonged antibiotic therapy [2,3,8]. Penicillin G remains the gold-standard treatment, administered intravenously at high doses for four to six weeks, followed by oral penicillin V or amoxicillin for six to 12 months [1-3]. Other effective antibiotics include erythromycin, streptomycin, clindamycin, cephalosporins, and tetracyclines [2,3]. These newer reports [6,7] confirm that prolonged therapy remains essential and provide additional data on outcomes in modern clinical practice [8]. CT and MRI are necessary tools to monitor therapeutic response [5,7,10].

CNS actinomycosis is a severe condition, with reported mortality rates ranging from 11% to 28%, and morbidity rates up to 54% [3,4,6,8]. A recent systematic review further emphasizes the severity and the importance of early diagnosis and treatment to improve patient outcomes, noting that delays in diagnosis remain a critical factor in prognosis [2].

## Conclusions

Cerebral actinomycosis is a rare and insidiously progressive infection, often diagnosed late due to its nonspecific clinical and radiological features. Imaging may mimic pseudotumoral lesions or other central nervous system infections, making clinical suspicion essential. Diagnosis relies on a high index of suspicion, even in patients without classic risk factors, and frequently requires histopathological confirmation, as microbiological identification is slow and challenging. Effective management depends on prolonged targeted antibiotic therapy, often combined with surgical intervention when indicated. Early recognition, appropriate combined therapy, and long-term neurological and functional follow-up are critical to improving outcomes.
